# Relationship between dietary flavan-3-ols intake and mortality in metabolic syndrome population; a large cohort study

**DOI:** 10.3389/fnut.2025.1572189

**Published:** 2025-04-10

**Authors:** Wanjia Zhang, Qian Zhou, Weiqing Yang, Xiaoqin Tan, Yin Xu, Zhan Yi

**Affiliations:** ^1^Graduate School, Hunan University of Chinese Medicine, Changsha, Hunan, China; ^2^Department of Gastroenterology, The First Hospital of Hunan University of Chinese Medicine, Changsha, Hunan, China; ^3^Xiangxing College, Hunan University of Chinese Medicine, Changsha, Hunan, China

**Keywords:** flavan-3-ols, metabolic syndrome, mortality, NHANES, cohort study, flavan-3-ol monomers

## Abstract

**Background:**

Metabolic syndrome (MetS) is a global health concern linked to increased mortality. Diets rich in plant-derived compounds, such as polyphenols, have shown potential health benefits for MetS. Among these, flavan-3-ols, a class of commonly occurring polyphenolic compounds, are known for their antioxidant and anti-inflammatory properties. Therefore, we hypothesize that flavan-3-ols intake is negatively associated with mortality risk in MetS population.

**Methods:**

This study analyzed NHANES data (2007–2008, 2009–2010, and 2017–2018). Flavan-3-ol and monomer intake were obtained from the USDA Flavonoid and FNDDS databases. Associations with mortality were assessed using Cox regression, survival differences were compared using Kaplan–Meier curves, and non-linear trends were examined using restricted cubic splines. Subgroup analyses were conducted to explore potential effect modifications.

**Results:**

Over a median follow-up period of 114 months, 1,856 participants survived, while 329 deaths were recorded. In Model 3, participants in the highest tertile (T3) of flavan-3-ol intake exhibited a 33% lower risk of all-cause mortality compared to those in the lowest tertile (T1) (HR = 0.67, 95%CI: 0.49–0.92). For monomers, the hazard ratios ranged from 0.55 for higher levels of epigallocatechin to 0.71 for higher levels of gallocatechin. Kaplan–Meier curves indicated significant differences in survival status across dietary flavan-3-ol intake groups. However, no association was found between flavan-3-ol intake and cardiovascular mortality risk. Additionally, restricted cubic spline (RCS) analysis did not reveal any non-linear relationship, and no significant interaction effects were observed in the subgroup analysis.

**Conclusion:**

Higher dietary intake of flavan-3-ols is negatively associated with mortality risk in MetS population.

## 1 Introduction

Metabolic Syndrome (MetS) is defined by a cluster of metabolic risk factors, including dyslipidemia, hypertension, central obesity, and insulin resistance (1). It has become a major global health concern, affecting approximately 20–25% of the world’s population, with a particularly high prevalence in high-income countries (2). According to the National Health and Nutrition Examination Survey (NHANES, 2011–2016), the prevalence of MetS in the U.S. population is estimated to be 34.7% (3). Additionally, MetS is frequently associated with a state of low-grade systemic inflammation, which further exacerbates insulin resistance and increases mortality risk (4, 5). The global burden of MetS-related mortality continues to rise annually, making it one of the leading causes of death worldwide (6, 7). Current treatment strategies primarily involve pharmacological interventions, including lipid-lowering, glucose-lowering, and antihypertensive medications. However, long-term use of these agents imposes a substantial burden on hepatic and renal function (8–11). As all components of MetS are sensitive to lifestyle changes, it remains the effective and safe strategy for managing MetS and preventing its complications.

To present, a few dietary patterns with anti-inflammatory qualities have made headway in the treatment of MetS (12, 13). In this context, the role of bioactive compounds from diet has been repeatedly emphasized (14). Flavonoids, a class of polyphenolic compounds abundantly found in plants, are well-known for their anti-inflammatory, antioxidant, and antidiabetic properties (15). They contain the following six components: Isoflavones, Anthocyanins, Flavan-3-ols, Flavanones, Flavonoids, and Flavanols. Flavan-3-ols, a major contributor to polyphenol intake, are predominantly found in green tea, cocoa, fruits, and red wine (16, 17). And flavan-3-ols are playing an increasingly important role in the development of dietary recommendations and reference intake values (18–20). They consist of six main monomers: Epicatechin 3-gallate (ECG), Epigallocatechin 3-gallate (EGCG), Epicatechin (EC), Epigallocatechin (EGC), Gallocatechin (GC), and Catechin (C). Recent epidemiological studies have confirmed that flavan-3-ols can help prevent the onset of chronic metabolic diseases and improve metabolic function to some extent (21–23). Extensive *in vitro* studies have demonstrated that flavan-3-ols and their monomers exhibit anti-inflammatory and antioxidant properties (24, 25). However, while these monomers show promising effects in cell and animal models, excessive intake of monomeric supplements in clinical settings may impose additional hepatic and renal burdens and cause gastrointestinal distress (26, 27). Current public health guidelines emphasize obtaining nutrients from diverse dietary sources rather than relying on isolated supplements (28). Additionally, flavan-3-ols have been associated with improvements in metabolic parameters, including blood pressure, glucose, and lipid levels. However, the relationship between their dietary intake and mortality risk remains inconclusive in survival cohort studies (29–31). The relationship between flavan-3-ols and mortality risk in MetS population is still under investigation.

In this study, we hypothesize that higher dietary intake of flavan-3-ols is negatively associated with mortality risk in individuals with MetS. To test this hypothesis, we utilized data from the USDA Flavonoid Database to estimate flavan-3-ol intake from various foods and beverages. Mortality outcomes were obtained from the National Death Index (NDI) death certificate records. Using the NHANES dataset, we conducted a comprehensive analysis to examine the association.

## 2 Methods

### 2.1 Research design

**Figure 1:**
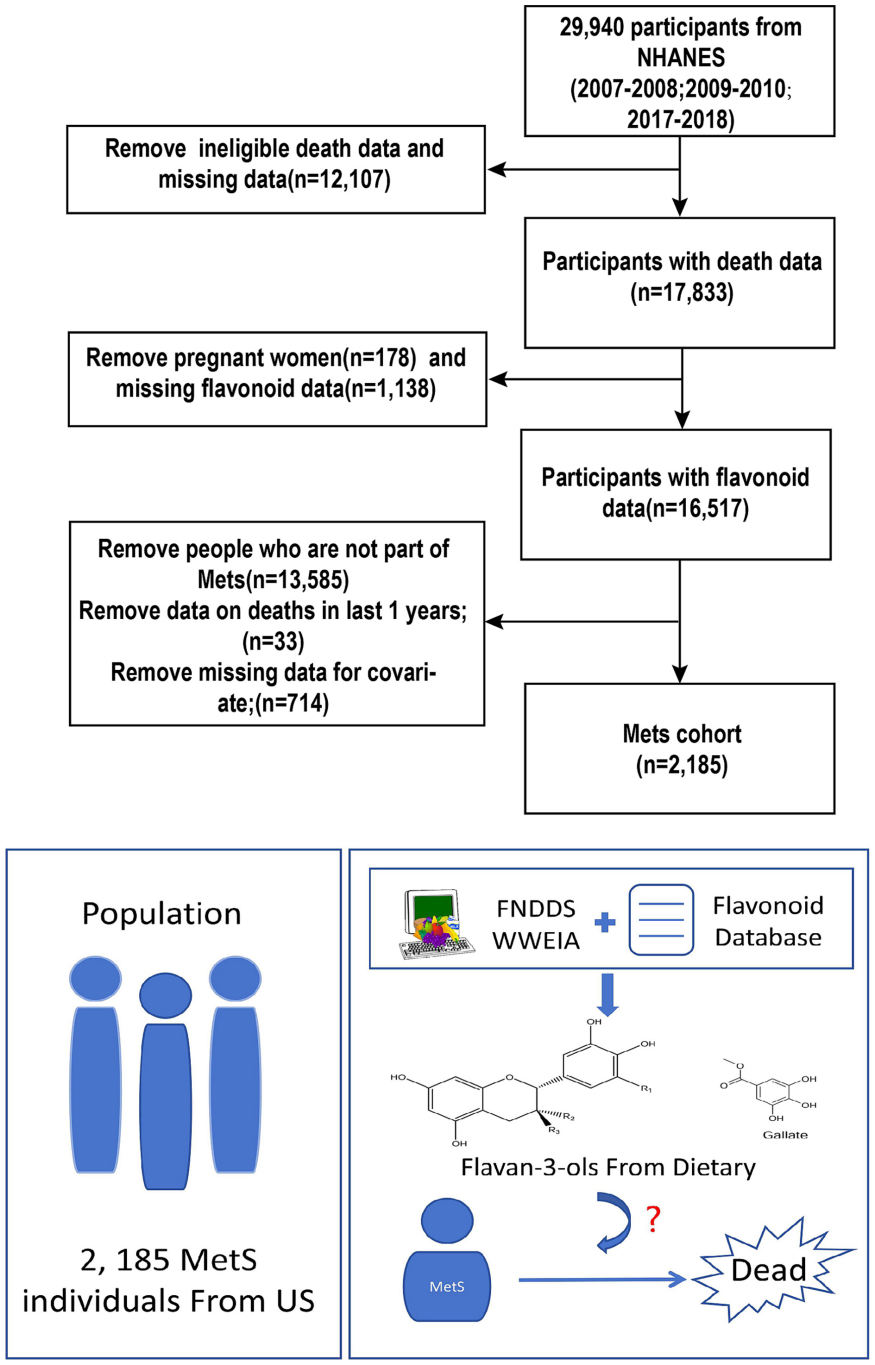
Flowchart of the study population.

The National Health and Nutrition Examination Survey (NHANES) is an important program that tracks nutrition and health trends in the U.S. population. All participants provide written consent, and the study undergoes strict ethical oversight (32). To ensure broad demographic representation, it employs a complex, multistage sampling framework. Comprehensive details on its methodology and publicly available datasets can be accessed through the official platform (32). We combined three cycles comprising 29,940 participants (2007–2008, 2009–2010, and 2017–2018). Participants with missing flavan-3-ols, ineligible death records, deaths within 1 year, pregnant women, and those with missing covariates were excluded. The final sample size was 2,185 participants. Figure 1 presents the survey flowchart.

### 2.2 Assessment of metabolic syndrome

The definition of MetS is based on the National Cholesterol Education Program (NCEP) (33). MetS is diagnosed if three of the following conditions: (1) waist circumference of ≥88 cm (women) and ≥102 cm (men), (2) hypertriglyceridemia (TG ≥150 mg/dL), (3) low high-density lipoprotein cholesterol (HDL < 40 mg/dL in men or < 50 mg/dL in women), (4) systolic blood pressure ≥ 130 mm Hg, diastolic ≥85 mm Hg or previous diagnosis of hypertension, or antihypertensive medication (5) Elevated fasting glucose (FPG) (FPG ≥ 5.6 mmol/L, or diagnosis of type 2 diabetes, or glycosylated hemoglobin >6.5%).

### 2.3 Assessment of flavan-3-ols and mortality data

The USDA Flavonoid Database provides comprehensive information on dietary flavonoid values across various foods and beverages. Detailed data can be accessed at https://www.ars.usda.gov/ARSUserFiles/80400535/Data/Flav/Flav3.3.pdf. This database includes the flavonoid content (mg/100 g) of all foods and beverages listed in the USDA Food and Nutrient Database for Dietary Studies (FNDDS), corresponding to applicable the What We Eat in America (WWEIA) survey and NHANES dietary data releases (34). The NHANES dietary data are collected through two non-consecutive 24-h dietary recall interviews. In this study, we included the following flavonoids: Epicatechin 3-gallate (ECG), Epigallocatechin 3-gallate (EGCG), Epicatechin (EC), Epigallocatechin (EGC), Gallocatechin (GC), and Catechin (C).

Mortality data were determined using NHANES participant information matched to National Death Index (NDI) death certificate data dated through December 31, 2019 (35). Cardiovascular mortality is included with death code information for (I00-I09, I11, I13, I20-I51, and I60-I69).

### 2.4 Covariates

Our analyses considered potential confounders, including age, race, gender, education, household income and poverty rate (PIR), BMI, depression, alcohol intake, smoking history, the Healthy Eating Index-2020 (HEI-2020) (36), history of cancer, and history of cardiovascular disease. Educational attainment was divided as </=/> High school. Race was categorized into five groups, including Mexican American, non-Hispanic Black and White, other Hispanic, or other race. PIR, an index measuring household socioeconomic status, was grouped as <1.5, 1.5–3.5, or >3.5. Depression was assessed using the Patient Health Questionnaire-9 (PHQ-9), a widely used screening tool for depressive symptoms, with a score of ≥10 indicating depression (37). Alcohol consumption history was categorized as never, moderate (<=2 drinks/day), and moderately heavy (>2 drinks/day). Smoking history was categorized as not smoking/quitting smoking and ongoing smoking. Cancer history was categorized as yes or no based on the patient’s questionnaire response “Have you ever been told by a doctor about any type of cancer.” Cardiovascular history included a history of stroke, angina, coronary heart disease, heart attack, and congestive heart failure as collected by the questionnaire. HEI-2020 assessed the diet of the U.S. population in 13 different areas (36): total vegetables, vegetables and legumes, total fruits, whole fruits, whole grains, seafood and plant proteins, dairy products, total protein foods, saturated fats, added sugars, fatty acids, refined grains, and sodium. The HEI-2020 is scored on a scale of 0–100, with higher scores reflecting healthier dietary patterns.

### 2.5 Statistical analysis

Dietary flavan-3-ols intake were stratified into tertiles, while individual monomers were categorized into tertiles or dichotomized based on their distributional characteristics to ensure robust and accurate analysis. To reduce outcome bias, death data within the first year of follow-up were excluded. Given the complex sampling design of the NHANES study, all analyses incorporated sample weights to maintain representativeness and validity.

Continuous data were presented as the mean (standard deviation), while binary variables were reported as unweighted frequencies (weighted frequencies). Between-group differences were assessed using the chi-square test for binary variables and the Wilcoxon rank-sum test for continuous variables. Kaplan–Meier curves were generated to evaluate survival across different levels of flavan-3-ols intake. The association between flavan-3-ols intake and mortality in MetS population was analyzed using a multivariate Cox regression model. To explore potential non-linear relationships, restricted cubic spline (RCS) regression was employed. Additionally, the analysis was stratified by age, sex, BMI, income, cancer history, and cardiovascular disease history, with interaction tests conducted to evaluate the presence of subgroup dependencies. All analyses were performed using R software version 4.2.3.

## 3 Results

### 3.1 Baseline information

The study included 2,185 participants with MetS, representing approximately 27 million Americans. Over a median follow-up of 114 months, 1,856 participants survived, while 329 deaths were recorded. The mean age of the cohort was 54.2 years, with males comprising 49% of the population. The average BMI was 32.8 kg/m^2^. Notably, significant differences were observed between groups for C, EGC, EC, ECG, EGCG, and GC. Participants who died during the study period were generally older, had lower education levels, lower income, lower BMI, and lower alcohol consumption, and exhibited a higher prevalence of cancer and cardiovascular disease. Detailed cohort characteristics are presented in Table 1, while the distribution of flavan-3-ols and their monomers is summarized in Supplementary Tables 1, 2. Supplementary Table 3 presents liver and kidney conditions across different flavan-3-ols intake groups, showing that the T3 group had lower ALT and AST levels, but no significant differences in liver diseases (fatty liver/viral hepatitis), serum creatinine, or chronic kidney disease (CKD) prevalence. US-FLI was used to assess fatty liver (38), and eGFR was calculated based on the CKD Epidemiology Collaboration (CKD-EPI) equation (39). Additionally, Supplementary Figure 1 illustrates the Pearson correlation coefficients among the major monomers.

**Table 1:** Characteristics of participants based on all-cause mortality.

Characteristic	Overall *N* = 27,429,291	Alive, *n* = 1856 (89%)	Death, *n* = 329 (11%)	*p*-value
Age (years)	54.20 (15.39)	52.41 (14.84)	69.00 (11.38)	<0.001
Race (%)				<0.001
Mexican American	368 (8.6%)	347 (9.3%)	21 (2.9%)	
Other Hispanic	198 (4.3%)	178 (4.5%)	20 (1.9%)	
Non-Hispanic White	1,096 (73%)	870 (71%)	226 (83%)	
Non-Hispanic Black	388 (9.2%)	330 (9.0%)	58 (11%)	
Other Race	135 (5.4%)	131 (5.8%)	4 (1.9%)	
Sex (%)				0.420
Male	1,033 (49%)	864 (50%)	169 (47%)	
Female	1,152 (51%)	992 (50%)	160 (53%)	
Education (%)				<0.001
<High School	626 (18%)	500 (17%)	126 (33%)	
High School	570 (29%)	472 (29%)	98 (31%)	
>High School	989 (52%)	884 (54%)	105 (36%)	
Family Income (%)				<0.001
Low	830 (26%)	686 (25%)	144 (36%)	
Medium	766 (34%)	637 (33%)	129 (41%)	
High	589 (39%)	533 (42%)	56 (22%)	
BMI (Kg/m^2^)	32.88 (6.25)	33.05 (6.24)	31.49 (6.20)	<0.001
Depression (%)				0.287
No	1943 (90%)	1,644 (90%)	299 (92%)	
Yes	242 (10.0%)	212 (10%)	30 (8.2%)	
Drinking (%)				<0.001
Never	827 (32%)	646 (29%)	181 (55%)	
Moderate	897 (47%)	788 (49%)	109 (35%)	
Heavy	461 (21%)	422 (22%)	39 (10%)	
Smoking (%)				0.987
No	1778 (83%)	1,510 (83%)	268 (83%)	
Yes	407 (17%)	346 (17%)	61 (17%)	
HEI-2020	50.14 (11.45)	50.00 (11.52)	51.33 (10.79)	0.072
Cancer History (%)				<0.001
No	1889 (87%)	1,648 (89%)	241 (72%)	
Yes	296 (13%)	208 (11%)	88 (28%)	
CVD History (%)				<0.001
No	1781 (85%)	1,588 (88%)	193 (60%)	
Yes	404 (15%)	268 (12%)	136 (40%)	
Flavan-3-ols (mg)	201.61 (398.70)	209.99 (409.38)	132.55 (287.78)	0.002
C (mg)	7.71 (8.92)	7.94 (9.17)	5.83 (6.24)	0.004
EGC (mg)	19.65 (46.47)	20.53 (48.17)	12.34 (27.79)	0.032
EC (mg)	10.35 (14.25)	10.63 (14.70)	8.01 (9.50)	0.015
ECG (mg)	12.58 (29.98)	13.13 (31.02)	8.06 (18.79)	0.002
EGCG (mg)	33.34 (94.71)	34.75 (98.74)	21.64 (49.07)	0.015
GC (mg)	1.96 (4.24)	2.05 (4.36)	1.24 (2.95)	0.003

BMI, Body Mass Index; CVD, history of cardiovascular disease; C, catechin; EGC, epigallocatechin; EC, epicatechin; ECG, epicatechin 3-gallate; EGCG, epigallocatechin 3-gallate; GC, gallocatechin; N, weighted population estimate; n, unweighted sample size.

### 3.2 The relationship between flavan-3-ols and all-cause mortality

Table 2 presents the results of the Cox regression analysis. Supplementary Table 4 displays the VIF values for variables in Model 3, with all VIF values below 5, indicating no significant multicollinearity. In Model 3, the T3 group of flavan-3-ols demonstrated a 33% reduction in the risk of all-cause Mortality compared to the T1 group (HR = 0.67, 95% CI: 0.49–0.92, P for trend = 0.01). Similarly, the T3 group of C exhibited a 43% lower risk of mortality compared to the T1 group (HR = 0.57, 95% CI: 0.39–0.84), and the T3 group of EGC showed a 45% reduction in mortality risk compared to the T1 group (HR = 0.55, 95% CI: 0.38–0.81).

**Table 2:** The relationship between flavan-3-ols intake and all-cause mortality.

Characteristic	Model 1	Model 2	Model 3	*P* for trend
	HR (95%CI) *p*-value	HR (95%CI) *p*-value	HR (95%CI) *p*-value	
Flavan-3-ols (mg)				0.01
T1 (<6.62)	ref	ref	ref	
T2 (6.62–56.88)	0.74 (0.54, 1.01)	0.73 (0.55, 0.98)*	0.91 (0.66, 1.25)	
T3 (>56.88)	0.58 (0.42, 0.79)**	0.60 (0.45, 0.79)**	0.67 (0.49, 0.92)*	
C (mg)				0.01
T1 (<2.52)	Ref	Ref	Ref	
T2 (2.52–7.79)	0.63 (0.45, 0.90)*	0.62 (0.43, 0.87)**	0.70 (0.47, 1.05)	
T3 (>7.79)	0.51 (0.37, 0.71)**	0.48 (0.33, 0.69)**	0.57 (0.39, 0.84)**	
EGC (mg)				0.01
T1 (<0.22)	Ref	Ref	Ref	
T2 (0.22–2.29)	0.87 (0.65, 1.16)	0.64 (0.47, 0.87)**	0.66 (0.48, 0.92)*	
T3 (>2.29)	0.63 (0.44, 0.91)*	0.54 (0.39, 0.74)**	0.55 (0.38, 0.81)**	
EC (mg)				0.31
T1 (<2)	Ref	Ref	Ref	
T2 (2–9.60)	0.94 (0.69, 1.30)	0.88 (0.68, 1.14)	0.93 (0.71, 1.23)	
T3 (>9.60)	0.62 (0.45, 0.86)**	0.69 (0.51, 0.93)*	0.80 (0.56, 1.13)	
ECG (mg)				NA
Group 1 (=0)	Ref	Ref	Ref	
Group 2 (>0)	0.65 (0.50, 0.85)**	0.59 (0.46, 0.77)**	0.66 (0.48, 0.89)**	
EGCG (mg)				NA
Group 1 (=0)	Ref	Ref	Ref	
Group 2 (>0)	0.72 (0.54, 0.95)*	0.63 (0.51, 0.79)**	0.70 (0.54, 0.90)**	
GC (mg)				NA
Group 1 (=0)	Ref	Ref	Ref	
Group 2 (>0)	0.60 (0.47, 0.77)**	0.63 (0.49, 0.82)**	0.71 (0.54, 0.95)*	

Model 1: non-adjusted.

Model 2: adjusted for age, sex, race, education, PIR.

Model 3: adjusted for age, sex, race, education, PIR, BMI, depression, drinking, smoking, HEI-2020, Cancer history, and cardiovascular history (Congestive heart failure, coronary heart disease, angina, heart attack, stroke).

C, catechin; EGC, epigallocatechin; EC, epicatechin; ECG, epicatechin 3-gallate; EGCG, epigallocatechin 3-gallate; GC, gallocatechin. *p < 0.5, **p < 0.01.

For ECG, participants in Group 2 (>0) had a 34% lower risk of mortality compared to Group 1 (=0) (HR = 0.66, 95% CI: 0.48–0.89). Similarly, Group 2 (>0) of EGCG was associated with a 30% reduction in mortality risk compared to Group 1 (=0) (HR = 0.70, 95% CI: 0.54–0.90), and Group 2 (>0) of GC had a 29% lower risk of mortality compared to Group 1 (=0) (HR = 0.71, 95% CI: 0.54–0.95). However, no significant differences in survival status were observed for EC.

### 3.3 The relationship between flavan-3-ols and cardiovascular mortality

Table 3 presents the results of the Cox regression analysis. In Model 1, participants in the T3 group of C had a 50% lower risk of mortality compared to those in the T1 group (HR = 0.50, 95% CI: 0.27–0.96). In Model 2, Group 2 of ECG showed a 40% lower risk of mortality compared to Group 1 (HR = 0.60, 95% CI: 0.41–0.88). However, in Model 3, no significant associations were observed between flavan-3-ols or monomers and cardiovascular mortality.

**Table 3:** The relationship between flavan-3-ols intake and cardiovascular mortality.

Characteristic	Model 1	Model 2	Model 3	*P* for trend
	HR (95%CI) *P*-value	HR (95%CI) *P*-value	HR (95%CI) *P*-value	
Flavan-3-ols (mg)				0.76
T1 (<6.62)	ref	ref	ref	
T2 (6.62–56.88)	0.86 (0.43, 1.69)	0.81 (0.45, 1.46)	1.01 (0.54, 1.87)	
T3 (>56.88)	0.80 (0.49, 1.30)	0.82 (0.51, 1.31)	0.94 (0.53, 1.67)	
C (mg)				0.14
T1 (<2.52)	Ref	Ref	Ref	
T2 (2.52–7.79)	0.71 (0.37, 1.33)	0.66 (0.35, 1.23)	0.72 (0.34, 1.51)	
T3 (>7.79)	0.50 (0.27, 0.96)*	0.45 (0.21, 0.93)*	0.54 (0.24, 1.20)	
EGC (mg)				0.95
T1 (<0.22)	Ref	Ref	Ref	
T2 (0.22–2.29)	1.17 (0.66, 2.08)	0.84 (0.43, 1.68)	0.82 (0.39, 1.72)	
T3 (>2.29)	1.01 (0.55, 1.83)	0.88 (0.46, 1.68)	0.89 (0.43, 1.86)	
EC (mg)				0.81
T1 (<2)	Ref	Ref	Ref	
T2 (2–9.60)	1.22 (0.78, 1.90)	1.14 (0.79, 1.65)	1.26 (0.82, 1.93)	
T3 (>9.60)	0.74 (0.38, 1.43)	0.82 (0.42, 1.60)	0.99 (0.49, 2.03)	
ECG (mg)				NA
Group 1 (=0)	Ref	Ref	Ref	
Group 2 (>0)	0.68 (0.45, 1.05)	0.60 (0.41, 0.88)**	0.70 (0.43, 1.12)	
EGCG (mg)				NA
Group 1 (=0)	Ref	Ref	Ref	
Group 2 (>0)	0.85 (0.56, 1.29)	0.73 (0.49, 1.09)	0.84 (0.49, 1.41)	
GC (mg)				NA
Group 1 (=0)	Ref	Ref	Ref	
Group 2 (>0)	0.84 (0.57, 1.24)	0.88 (0.58, 1.33)	0.99 (0.62, 1.56)	

Model 1: non-adjusted.

Model 2: adjusted for age, sex, race, education, PIR.

Model 3: adjusted for age, sex, race, education, PIR, BMI, depression, drinking, smoking, HEI-2020, Cancer history, and cardiovascular history (congestive heart failure, coronary heart disease, angina, heart attack, stroke).

C, catechin; EGC, epigallocatechin; EC, epicatechin; ECG, epicatechin 3-gallate; EGCG, epigallocatechin 3-gallate; GC, gallocatechin. *P < 0.5, **P < 0.01.

### 3.4 Kaplan–Meier survival analysis

**Figure 2:**
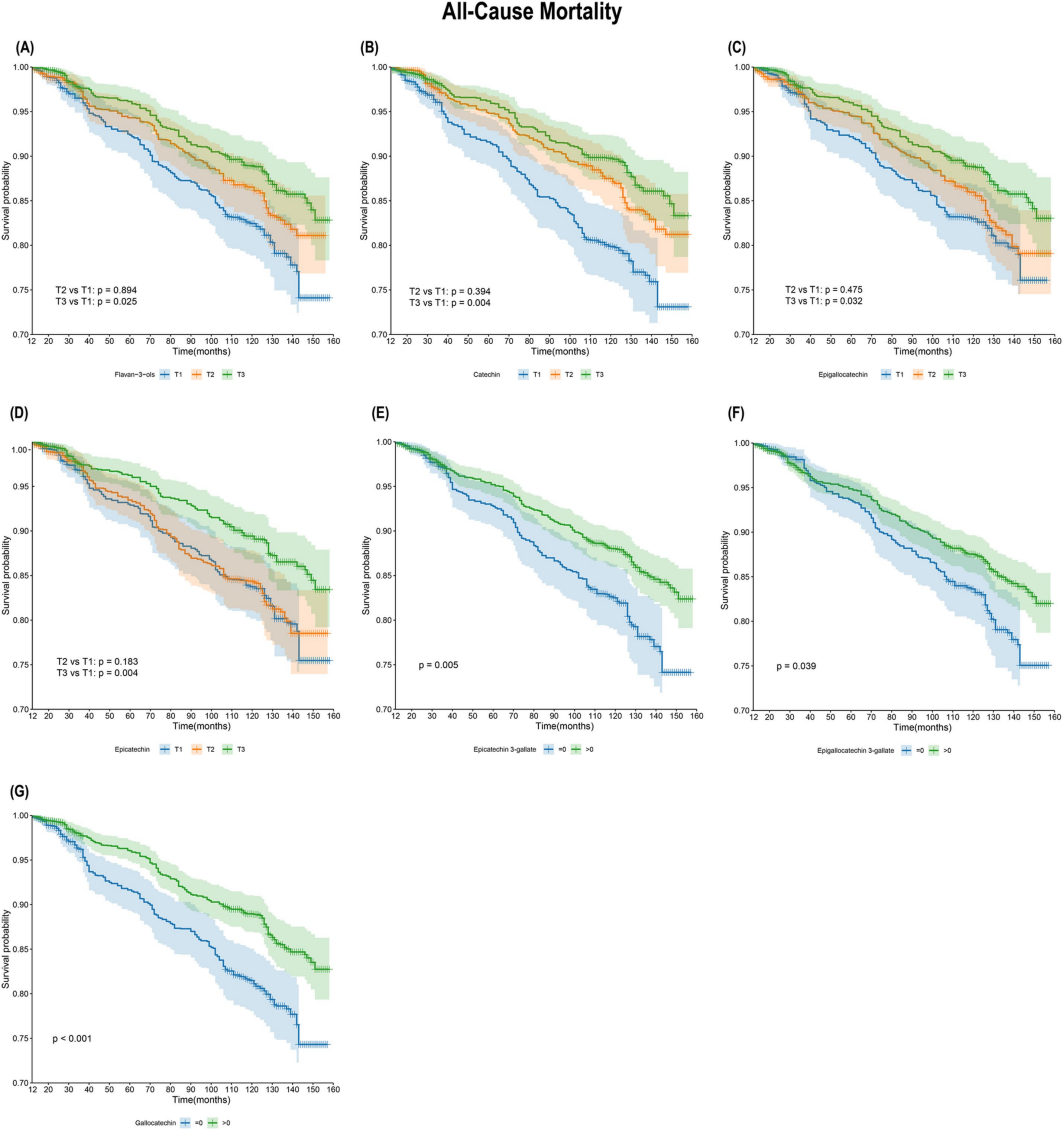
Kaplan–Meier survival analysis of flavan-3-ols intake and all-cause mortality. **(A)** Flavan-3-ols and all-cause mortality; **(B)** C and all-cause mortality; **(C)** EGC and all-cause mortality; **(D)** EC and all-cause mortality; **(E)** ECG and all-cause mortality; **(F)** EGCG and all-cause mortality; **(G)** GC and all-cause mortality.

As shown in Figure 2, KM survival curves were plotted for different flavan-3-ol or monomers, including flavan-3-ols, C, EGC, EC, ECG, EGCG, and GC. For all-cause Mortality, the results indicated that the survival rate of the T3 group was significantly higher than that of the T1 group for flavan-3-ols, C, EGC, and EC (log-rank test *p* < 0.05). Additionally, the survival rate of Group 2 (>0) was higher than that of Group 1 (=0) for ECG, EGCG, and GC. The relationship between flavan-3-ols and their monomers with Cardiovascular Mortality risk is further illustrated in Supplementary Figure 2. However, no significant differences in cardiovascular mortality were observed across different dietary intake groups of flavan-3-ols.

### 3.5 RCS analysis of flavan-3-ols and risk of all-cause mortality

**Figure 3:**
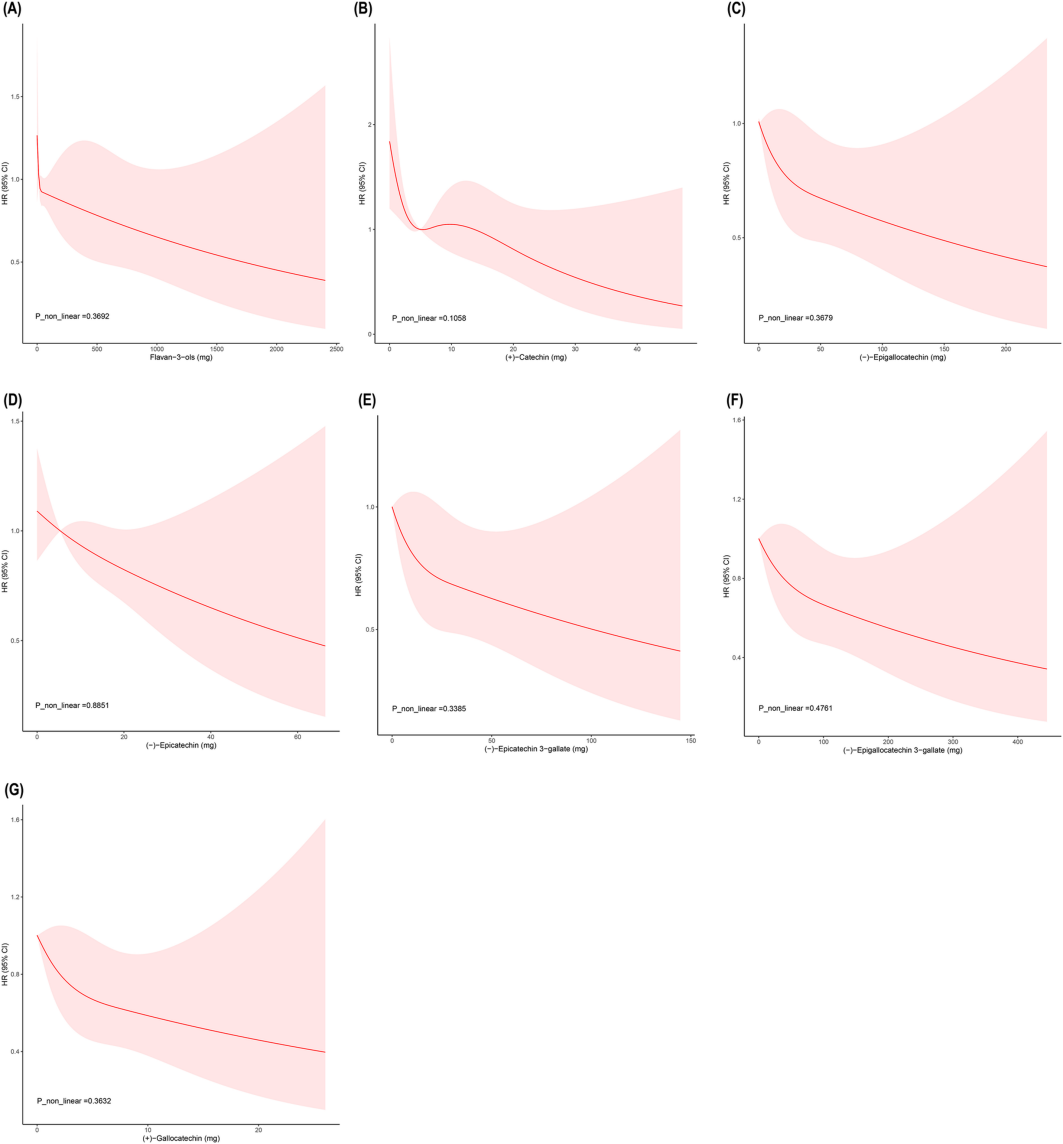
Dose–response relationship between flavan-3-ols and all-cause mortality. **(A)** Flavan-3-ols; **(B)** C; **(C)** EGC; **(D)** EC; **(E)** ECG; **(F)** EGCG; **(G)** GC.

To further explore the potential dose–response relationship between flavan-3-ols and all-cause Mortality, we employed restricted cubic spline (RCS) analysis. As illustrated in Figure 3, the statistical tests did not reveal any significant non-linear association (*p* > 0.05). This could imply that the protective effect of flavan-3-ols against all-cause Mortality may be dose-dependent, with higher levels of intake potentially contributing to a reduced mortality risk.

### 3.6 Subgroup analysis, interaction test and sensitivity analysis

Table 4 presents the results of the subgroup analysis on flavan-3-ols and all-cause mortality. The T3 group of flavan-3-ols was negatively associated with all-cause mortality in MetS individuals aged >60 years (HR = 0.65, 95% CI: 0.44–0.98), in the female MetS population (HR = 0.63, 95% CI: 0.42–0.92), and at the low-income level (HR = 0.63, 95% CI: 0.42–0.94). Supplementary Table 5 presents the association between flavan-3-ols and cardiovascular mortality across different subgroups. However, no significant interactions were found between flavan-3-ols and these subgroups in all interaction tests (*p* > 0.05 for interaction). Notably, no significant differences in prognosis were observed across different flavan-3-ols intake levels among individuals with varying liver and kidney conditions (*p* > 0.05 for interaction).

**Table 4:** Subgroup analysis and interaction test between flavan-3-ols and all-cause mortality.

Characteristic	Number	T1	T2	T3	*P* for interaction
		HR (95%CI) *P*-value	HR (95%CI) *P*-value	HR (95%CI) *P*-value	
Age					0.874
20–60	1,182	Ref	0.81 (0.35,1.87)	0.57 (0.27,1.22)	
61–80	1,003	Ref	0.92 (0.60,1.40)	0.65 (0.44,0.98)*	
Sex					0.866
Male	1,033	Ref	1.13 (0.67,1.92)	0.78 (0.45,1.38)	
Female	1,152	Ref	0.77 (0.53,1.13)	0.63 (0.42,0.92)*	
Family income					0.907
Low	830	Ref	0.79 (0.44,1.42)	0.63 (0.42,0.94)*	
Medium	766	Ref	0.80 (0.53,1.21)	0.61 (0.37,1.02)	
High	589	Ref	1.16 (0.42,3.25)	0.54 (0.18,1.60)	
BMI					0.257
<30	844	Ref	0.98 (0.70,1.38)	0.69 (0.44,1.10)	
>=30	1,341	Ref	0.82 (0.48,1.41)	0.68 (0.45,1.02)	
Cancer					0.716
No	1889	Ref	1.06 (0.70,1.60)	0.72 (0.46,1.12)	
Yes	296	Ref	0.85 (0.43,1.68)	0.51 (0.27,0.97)*	
CVD					0.700
No	1781	Ref	0.90 (0.64,1.27)	0.75 (0.51,1.10)	
Yes	404	Ref	0.89 (0.47,1.70)	0.56 (0.31,1.00)	
Liver diseases					0.972
No	759	Ref	0.98 (0.48,1.96)	0.67 (0.43,1.05)	
Yes	1,414	Ref	0.90 (0.54,1.49)	0.66 (0.42,1.02)	
CKD					0.658
No	1911	Ref	0.98 (0.63,1.53)	0.67 (0.44,1.02)	
Yes	262	Ref	0.62 (0.36,1.08)	0.58 (0.34,1.00)	

BMI, Body Mass Index; CVD, history of cardiovascular disease; Liver diseases: includes diagnoses of fatty liver disease or viral hepatitis; CKD, chronic kidney disease, defined as an estimated glomerular filtration rate (eGFR) < 60 mL/min per 1.73 m^2^. US-FLI was used to assess fatty liver (38). The eGFR was calculated with reference to the recommendations of the Epidemiology of CKD (39). *P < 0.5, **P < 0.01.

To address potential bias introduced by the discontinuity in the time series, we conducted a sensitivity analysis by excluding the 2017–2018 data and reanalyzing the dataset. The results in Supplementary Table 6 and Supplementary Figure 3 remained consistent with our primary findings, reinforcing the robustness of our conclusions.

## 4 Discussion

In this study, we extracted data from the FNDDS and NHANES databases to investigate the relationship between flavan-3-ols intake and mortality risk, which included 2,185 MetS population. Our findings reveal that higher intake of flavan-3-ols are negatively associated with all-cause Mortality risk, and this protective effect may be a dose-dependent relationship. This negative correlation was consistent across most flavan-3-ol monomers. KM analysis further showed that individuals with higher flavan-3-ols intake had a significantly better survival rate compared to those with lower intake. However, no significant association was found between flavan-3-ol intake and the risk of cardiovascular mortality.

It is well known that the main sources of flavan-3-ols include red wine, green tea, cocoa, and fruits. Mei Chung and colleagues synthesized 39 prospective cohorts and found that an increase of one cup (236.6 mL) of tea (the main source of flavan-3-ol monomers) per day was related to a 1.5% lower risk of all-cause mortality and a 4% lower cardiovascular risk (40). A Nurses’ Health Study II (NHS II) also demonstrated that the consumption of red wine, tea, and fruits is inversely associated with the risk of all-cause mortality (29). Bondonno et al. (41) found that the protective effect of flavan-3-ols were not significant in the general population. However, they found a 25% reduction in risk of all-cause mortality in the highest tertile of flavan-3-ols compared with the lower tertile in an established subgroup of “high-risk individuals” (smokers, alcoholics, obese) (41). Our findings further support this observation, showing that even after adjusting for potential confounders such as alcohol consumption, smoking, and BMI, individuals in the T3 group of flavan-3-ol intake exhibited a 33% lower risk of all-cause mortality compared to those in the T1 group for MetS population. Notably, individuals with unhealthy habits or metabolic disorders often experience elevated levels of oxidative stress and inflammation, which predispose them to higher mortality risk. Given the potent antioxidant and anti-inflammatory properties of flavan-3-ols, their increased intake may provide substantial benefits to these at-risk populations. These results underscore the potential role of flavan-3-ols as a valuable dietary component for reducing mortality risk and improving health outcomes, particularly in metabolically compromised individuals.

To our knowledge, this is the first long-term survival cohort study about dietary flavan-3-ols in a MetS population. This study demonstrated that the protective effect of flavan-3-ols were significant in a high-risk population. Pro-inflammatory factors, including tumor necrosis factor-alpha (TNF-*α*), leptin, and interleukin-6 (IL-6), along with elevated levels of free fatty acids and adipokines, were markedly increased in individuals with MetS. These factors contributed to a heightened inflammatory state and oxidative stress, which elevated the risk of mortality (4, 5). In addition, macrophage polarization, thrombosis, monocyte adhesion, decreased nitric oxide utilization, vascular remodeling and stiffness, and oxidative stress levels have all been shown to play important roles in pathological mechanisms in metabolically disturbed populations (42–46). Flavan-3-ols can provide electrons to stop ROS production and chelate metal ions associated with free radical production (47). It also upregulate the activities of various ROS-scavenging enzymes, including glutathione peroxidase (GSH), superoxide dismutase (SOD), and catalase (CAT) (24, 25). They significantly reduced the secretion of inflammatory cytokines, including TNF-*α*, IL-6, and interleukin-1 beta (IL-1β), suppressing the activation of nuclear factor kappa B (NF-κB) *in vivo* and *in vitro*. Additionally, they exerted antioxidant effects through the activation of the nuclear factor erythroid 2-related factor 2 (Nrf2) pathway (48, 49).

Various monomers have also been extensively shown to improve the internal environment of MetS. EGCG has been widely shown to have preventive effects on various metabolic diseases (50). It modulates lipid absorption, increases glucose uptake and significantly improves symptoms of MetS. Supplementation with C in rats attenuated the expression of adipose inflammation (51). GC was shown to down-regulate 11 pro-inflammatory genes, including S100a8 and IL-1β, and up-regulate the expression of Gstm3 antioxidant genes in mouse experiments (52). Our analysis supplements the understanding of the long-term benefits of dietary flavan-3-ol monomers intake for MetS. In addition, we also found higher dietary intake including EGC, ECG, and EC were also protective against long-term survival states. It is important that although most monomers contribute positively to metabolic function, their strong correlation through dietary intake makes it difficult to pinpoint any single monomer as having a predominant effect.

Although some studies have emphasized the protective effects of antioxidant supplementation in mitigating the cardiovascular disease pathology in MetS population (53), the United States Pharmacopeia has issued warnings regarding the potential hepatotoxicity and gastrointestinal side effects of common monomeric extracts such as C, EGC, EC, ECG, EGCG, and GC (54). These adverse effects exhibit considerable individual variability and are closely linked to extraction methods and storage conditions. In contrast, dietary intake offers a safer alternative, avoiding the potential toxicity and risks associated with isolated supplements while fulfilling daily nutritional requirements (28). Recent dietary guidelines on flavan-3-ols confirm that an intake of 400–600 mg/day can improve heart metabolism, including blood pressure, blood glucose, and lipid levels (18). Our study further supports the benefits of increased flavan-3-ols consumption through diet for MetS population. The dose–response relationship in this study showed a linear relationship, suggesting that the risk of all-cause mortality may be negatively associated with flavan-3-ols dietary intake in the MetS population. Nevertheless, the clinical significance of flavan-3-ols intake requires further investigation, as individual metabolic differences and dietary variations may influence its protective effects. Future research should aim to determine an optimal intake threshold, providing a foundation for more precise dietary guidelines tailored to the MetS population. Establishing such recommendations could help maximize flavan-3-ols’potential in improving long-term health outcomes.

The program has several additional strengths. We included patient data from 3 cycles and considered corresponding weights to represent the broader US population. In addition, we adjusted for multiple confounding variables and factors and designed a rigorous analytic process to make the results more reliable.

However, this study has some limitations. Dietary intake of flavan-3-ols was collected in a baseline data sheet, and dietary habits fluctuated during long-term follow-up. Additionally, 24-h dietary recall, although widely used in large-scale epidemiological studies, may introduce recall bias and measurement errors. Furthermore, there may be some variation in the dietary composition of different populations, making our results of limited representativeness.

Although we adjusted for several confounding variables, we could not exclude all confounding factors. In addition, for all observational studies, we were unable to derive causal effects. The MetS population is at a higher risk of cardiovascular events, yet our study did not identify a significant association between flavan-3-ol intake and cardiovascular mortality risk. This may be attributable to sample size limitations, underscoring the need for further large-scale studies to explore this relationship in greater depth. Moreover, our study primarily focused on the association between flavan-3-ol intake and mortality, without an in-depth investigation into the underlying biological mechanisms. Future research also should incorporate relevant metabolic and inflammatory biomarkers to explore their potential mediating effects on this association.

## 5 Conclusion

In this large-scale U.S. population study, higher dietary intake of flavan-3-ols is negatively associated with mortality risk in MetS population. We believe that further research into the optimal dietary flavan-3-ols intake could provide significant benefits in reducing mortality risk.

## Data Availability

Publicly available datasets were analyzed in this study. This data can be found at: NHANES (https://www.cdc.gov/nchs/nhanes/index.htm).
