# Supplementary material for: Relationship between dietary flavan-3-ols intake and mortality in metabolic syndrome population; a large cohort study
Source: Front Nutr. 2025 Apr 10;12:1572189. doi: 10.3389/fnut.2025.1572189 (PMC12019991; doi:10.3389/fnut.2025.1572189)
Supplement: Supplementary file 1 [file Table_1.DOCX]

**Supplementary Table 1.** Distributions and concentrations of dietary Flavan-3-ols intakes (mg/day) in our research.

| **Flavan-3-ols class** | **5^th^** | **25^th^** | **50^th^** | **75^th^** | **95^th^** |
| --- | --- | --- | --- | --- | --- |
| Flavan-3-ols (mg) | 0.286 | 4.19 | 15.155 | 163.5 | 783.294 |
| C (mg) | 0.015 | 1.54 | 4.945 | 9.68 | 21.372 |
| EGC (mg) | 0 | 0.125 | 0.49 | 14.775 | 78.866 |
| EC (mg) | 0.061 | 1.065 | 5.335 | 12.575 | 29.974 |
| ECG (mg) | 0 | 0 | 0.025 | 9.68 | 50.992 |
| EGCG (mg) | 0 | 0 | 0.145 | 24.02 | 135.793 |
| GC (mg) | 0 | 0 | 0.015 | 1.49 | 7.886 |

**Annotate:** C:(+)-Catechin; EGC:(-)-Epigallocatechin; EC:(-)-Epicatechin; ECG:(-)-Epicatechin 3-gallate; EGCG:(-)-Epigallocatechin 3-gallate; GC:(+)-Gallocatechin;

5th, 5th percentile; 25th, 25th percentile; 50th, 50th percentile; 75th, 75th percentile; 95th, 95th percentile.


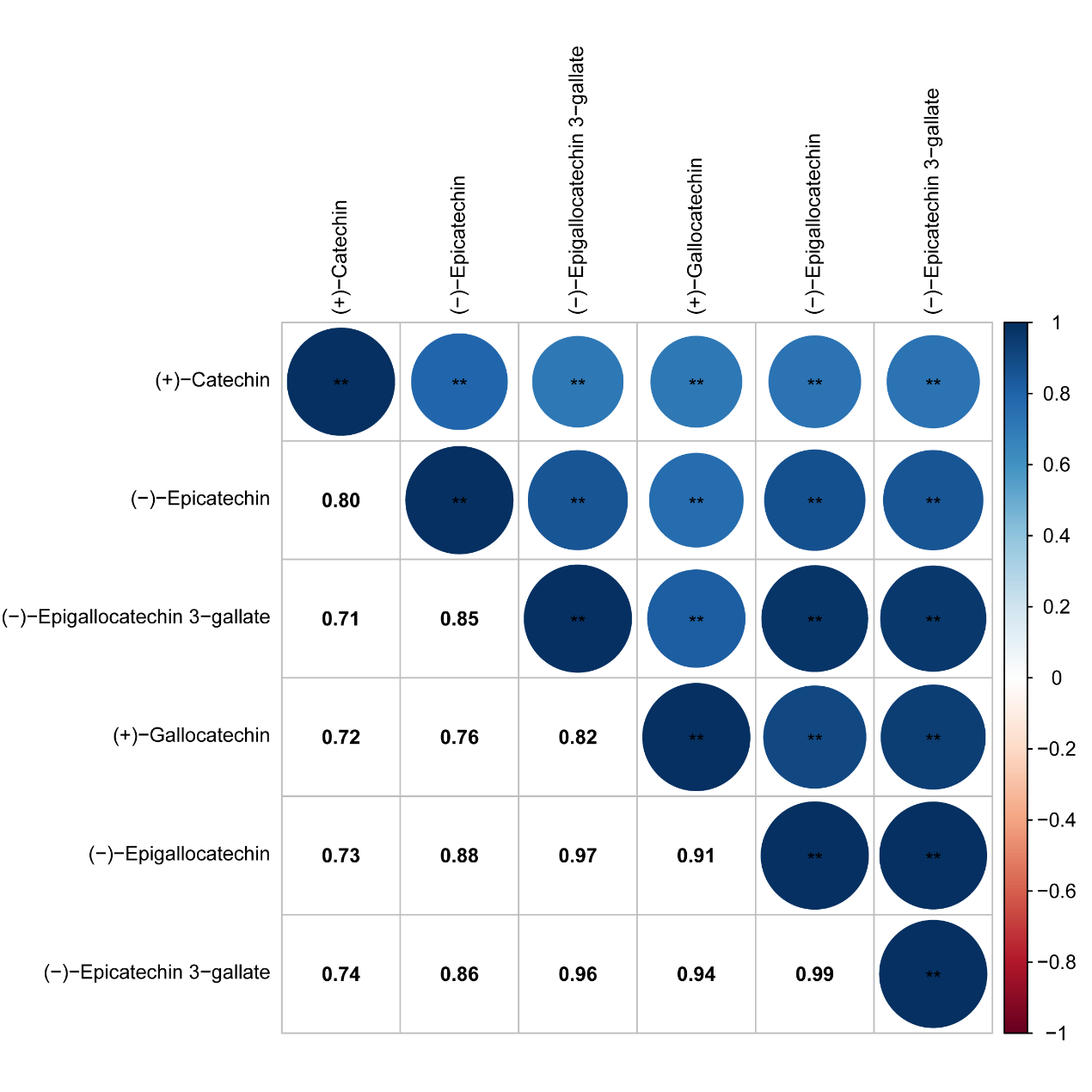


**Figure S1**. Pearson correlation coefficients among main monomers from dietary.

**Supplementary Table 2.** Comparison of dietary Flavan-3-ols intake (mg/day) among tertile groups(Flavan-3-ols) in our research.

| **Flavan-3-ols class** | **T1**  **n = 729 (30%)** | **T2**  **n = 728 (32%)** | **T3**  **n = 728 (38%)** | **P-value** |
| --- | --- | --- | --- | --- |
| Flavan-3-ols (mg) | 2.63 (1.98) | 17.90 (9.99) | 514.60 (511.25) | <0.001 |
| C (mg) | 1.32 (1.33) | 7.65 (4.97) | 12.88 (11.34) | <0.001 |
| EGC (mg) | 0.20 (0.24) | 0.68 (1.03) | 51.08 (64.07) | <0.001 |
| EC (mg) | 1.03 (1.08) | 8.11 (6.03) | 19.69 (18.53) | <0.001 |
| ECG (mg) | 0.01 (0.04) | 0.11 (0.35) | 33.08 (41.16) | <0.001 |
| EGCG (mg) | 0.02 (0.08) | 0.36 (1.27) | 87.59 (137.58) | <0.001 |
| GC (mg) | 0.05 (0.14) | 0.12 (0.29) | 5.03 (5.67) | <0.001 |

**Annotate:** C:(+)-Catechin; EGC:(-)-Epigallocatechin; EC:(-)-Epicatechin; ECG:(-)-Epicatechin 3-gallate; EGCG:(-)-Epigallocatechin 3-gallate; GC:(+)-Gallocatechin; Mean (SD);

**Supplementary Table 3.** Hepatic and renal function characteristics of participants based on flavan-3-ols intake.

| **Characteristic** | **T1**  **n = 729 (30%)** | **T2**  **n = 728 (32%)** | **T3**  **n = 728 (38%)** | **P-value** |
| --- | --- | --- | --- | --- |
| ALT | 27.89 (19.51) | 30.10 (23.70) | 26.11 (14.82) | 0.011 |
| AST | 24.92 (14.57) | 27.38 (19.29) | 24.13 (8.84) | 0.025 |
| SCR | 0.91 (0.32) | 0.90 (0.26) | 0.90 (0.38) | 0.724 |
| Liver Diseases |  |  |  | 0.188 |
| No | 249 (35%) | 246 (31%) | 275 (37%) |  |
| Yes | 480 (65%) | 482 (69%) | 453 (63%) |  |
| CKD |  |  |  | 0.164 |
| No | 628 (89%) | 645 (91%) | 650 (92%) |  |
| Yes | 101 (11%) | 83 (8.5%) | 78 (7.5%) |  |

**Annotate:** ALT: Alanine aminotransferase; AST: Aspartate aminotransferase; SCR: Serum creatinine; Liver diseases: Includes diagnoses of fatty liver or viral hepatitis; CKD: Chronic kidney disease, defined as an estimated glomerular filtration rate (eGFR) < 60 mL/min per 1.73 m². US-FLI was used to assess fatty liver(1); The eGFR was calculated with reference to the recommendations of the Epidemiology of CKD(2).

**Supplementary Table 4.** Variance Inflation Factor (VIF) values for all variables.

| **Variable** | **VIF** |
| --- | --- |
| Flavan-3-ols | 1.766 |
| Race | 1.498 |
| Age | 1.93 |
| Sex | 2.145 |
| Education | 1.964 |
| Family Income | 2.056 |
| Depression | 1.525 |
| Drinking | 2.002 |
| Smoking | 2.081 |
| HEI-2020 | 1.549 |
| CVD | 1.609 |
| BMI | 1.983 |
| Cancer History | 2.131 |

Annotate: BMI: Body Mass Index; CVD: History of cardiovascular disease;


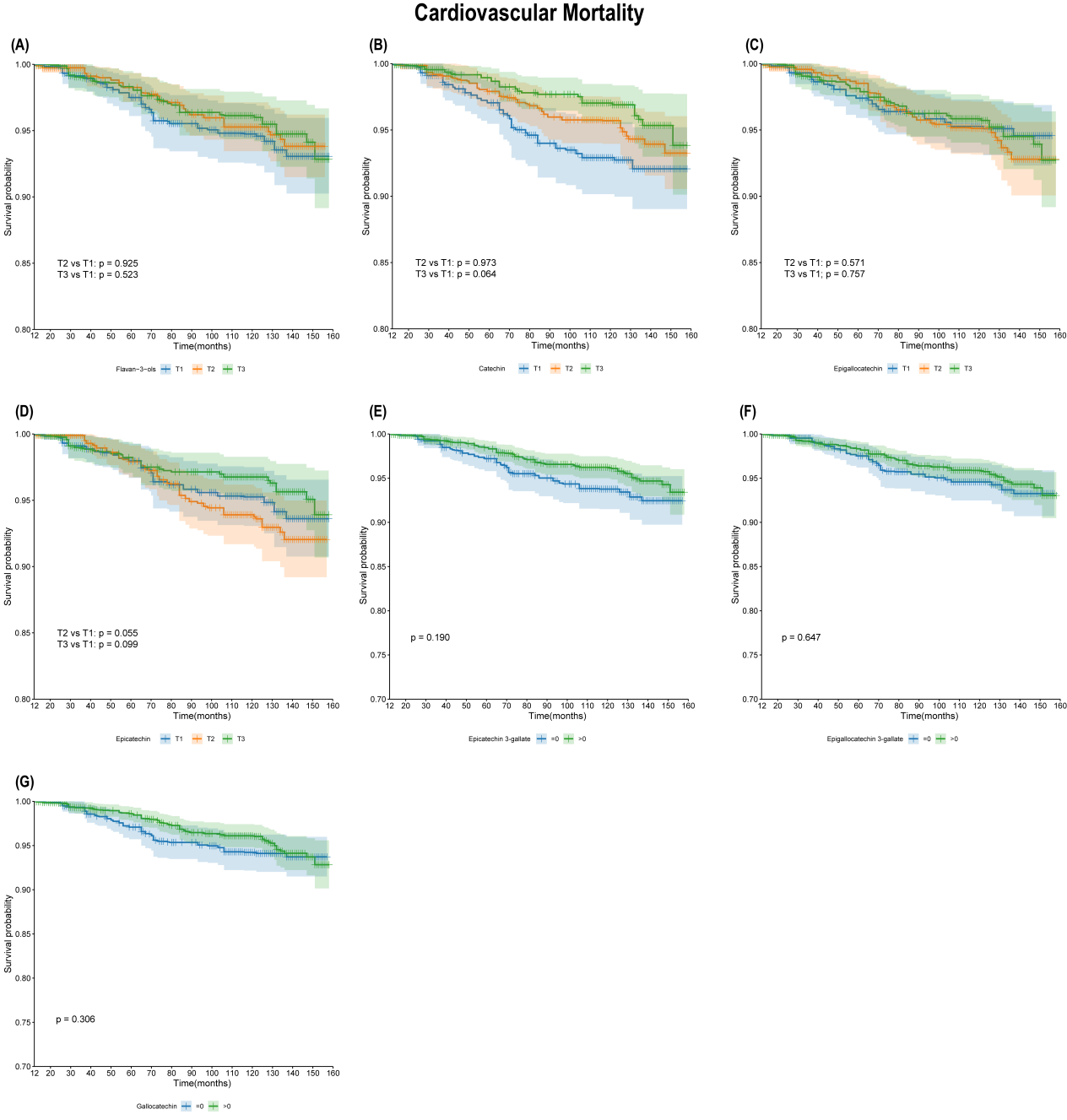


**Figure S2.** Kaplan-Meier Survival Analysis of Flavan-3-ols Intake and Cardiovascular Mortality. (A)Flavan-3-ols and Cardiovascular Mortality; (B)C and Cardiovascular Mortality; (C)EGC and Cardiovascular Mortality; (D) EC and Cardiovascular Mortality; (E)ECG and Cardiovascular Mortality; (F)EGCG and Cardiovascular Mortality; (G)GC and Cardiovascular Mortality.

**Supplementary Table 5.** Subgroup analysis and interaction test between Flavan-3-ols and Cardiovascular Mortality

| Characteristic | Number | T1 | T2 | T3 | P for interaction |
| --- | --- | --- | --- | --- | --- |
|  |  | HR(95%CI) | HR(95%CI) | HR(95%CI) |  |
| **Age** |  |  |  |  | 0.519 |
| 20-60 | 1182 | Ref | 0.51  (0.13,2.07) | 1.00  (0.29,3.39) |  |
| 61-80 | 1003 | Ref | 1.01  (0.47,2.18) | 0.78  (0.42,1.48) |  |
| **Sex** |  |  |  |  | 0.884 |
| Male | 1033 | Ref | 1.24  (0.50,3.08) | 0.88  (0.31,2.47) |  |
| Female | 1152 | Ref | 0.73  (0.27,1.97) | 0.93  (0.44,1.96) |  |
| **Family Income** |  |  |  |  | 0.952 |
| Low | 830 | Ref | 0.52  (0.22,1.20) | 0.58  (0.28,1.21) |  |
| Medium | 766 | Ref | 1.07  (0.51,2.24) | 0.85  (0.36,2.01) |  |
| High | 589 | Ref | 1.57  (0.27,9.26) | 1.37  (0.18,10.5) |  |
| **BMI** |  |  |  |  | 0.807 |
| <30 | 844 | Ref | 1.22  (0.40,3.76) | 1.19  (0.44,3.17) |  |
| >=30 | 1341 | Ref | 0.99  (0.47,2.08) | 0.85  (0.40,1.83) |  |
| **Cancer** |  |  |  |  | 0.758 |
| No | 1889 | Ref | 1.01  (0.51,2.00) | 0.97  (0.52,1.80) |  |
| Yes | 296 | Ref | 1.43  (0.23,8.85) | 1.03  (0.26,4.08) |  |
| **CVD** |  |  |  |  | 0.271 |
| No | 1781 | Ref | 0.78  (0.36,1.69) | 0.98  (0.46,2.09) |  |
| Yes | 404 | Ref | 1.31  (0.55,3.13) | 0.95  (0.41,2.19) |  |
| **Liver Diseases** |  |  |  |  | 0.268 |
| No | 759 | Ref | 0.57  (0.18,1.81) | 0.81  (0.39,1.69) |  |
| Yes | 1414 | Ref | 1.58  (0.70,3.53) | 1.15  (0.51,2.57) |  |
| **CKD** |  |  |  |  | 0.475 |
| No | 1911 | Ref | 1.32  (0.62,2.82) | 1.03  (0.50,2.12) |  |
| Yes | 262 | Ref | 0.48  (0.17,1.33) | 0.57  (0.19,1.71) |  |

Annotate: BMI: Body Mass Index; CVD: History of cardiovascular disease; Liver diseases: Includes diagnoses of fatty liver or viral hepatitis; CKD: Chronic kidney disease, defined as an estimated glomerular filtration rate (eGFR) < 60 mL/min per 1.73 m². US-FLI was used to assess fatty liver(1); The eGFR was calculated with reference to the recommendations of the Epidemiology of CKD(2). *P<0.5; **P<0.01

**Supplementary Table 6.** The relationship between flavan-3-ols intake and all-cause mortality(NHANES 2007-2010)

|  | Model 1 | Model 2 | Model 3 | P for trend |
| --- | --- | --- | --- | --- |
|  | HR (95%CI)  P-value | HR (95%CI)  P-value | HR (95%CI)  P-value |  |
| **Flavan-3-ols (mg)** |  |  |  | **0.01** |
| T1(<6.62) | ref | ref | ref |  |
| T2(6.62-43.01) | 0.77 (0.55, 1.06) | 0.75 (0.55, 1.01) | 0.93 (0.67, 1.31) |  |
| T3(>43.01) | 0.60 (0.44, 0.82)** | 0.60 (0.46, 0.79)** | 0.69 (0.50, 0.94)* |  |
| **C (mg)** |  |  |  | **0.01** |
| T1(<2.52) | Ref | Ref | Ref |  |
| T2(2.52-7.79) | 0.60 (0.42, 0.87)** | 0.58 (0.42, 0.81)** | 0.67 (0.46, 0.98)* |  |
| T3(>7.79) | 0.52 (0.37, 0.72)** | 0.48 (0.33, 0.67)** | 0.58 (0.40, 0.83)** |  |
| **EGC (mg)** |  |  |  | **0.01** |
| T1(<0.22) | Ref | Ref | Ref |  |
| T2(0.22-2.29) | 0.82 (0.59, 1.13) | 0.63 (0.45, 0.86)** | 0.65 (0.46, 0.92)* |  |
| T3(>2.29) | 0.63 (0.44, 0.90)* | 0.54 (0.39, 0.74)** | 0.55 (0.38, 0.81)** |  |
| **EC (mg)** |  |  |  | **0.10** |
| T1(<2) | Ref | Ref | Ref |  |
| T2(2-9.60) | 0.89 (0.64, 1.22) | 0.84 (0.67, 1.05) | 0.91 (0.72, 1.16) |  |
| T3(>9.60) | 0.56 (0.41, 0.75)** | 0.62 (0.45, 0.85)** | 0.72 (0.49, 1.06) |  |
| **ECG (mg)** |  |  |  | **NA** |
| Group 1(=0) | Ref | Ref | Ref |  |
| Group 2(>0) | 0.65 (0.50, 0.85)** | 0.59 (0.46, 0.77)** | 0.66 (0.49, 0.90)** |  |
| **EGCG (mg)** |  |  |  | **NA** |
| Group 1(=0) | Ref | Ref | Ref |  |
| Group 2(>0) | 0.72 (0.54, 0.95)* | 0.63 (0.50, 0.78)** | 0.70 (0.54, 0.92)* |  |
| **GC (mg)** |  |  |  | **NA** |
| Group 1(=0) | Ref | Ref | Ref |  |
| Group 2(>0) | 0.59 (0.46, 0.76)** | 0.62 (0.48, 0.80)** | 0.71 (0.53, 0.95)* |  |

Model 1: non-adjusted

Model 2: adjusted for age, sex, race, education, PIR;

Model 3: adjusted for age, sex, race, education, PIR, BMI, depression, drinking, smoking, HEI-2020, Cancer history, and cardiovascular history (Congestive heart failure, coronary heart disease, angina, heart attack, stroke);

Annotate: C: Catechin; EGC: Epigallocatechin; EC: Epicatechin; ECG: Epicatechin 3-gallate; EGCG: Epigallocatechin 3-gallate; GC: Gallocatechin; *:P<0.5; **:P<0.01


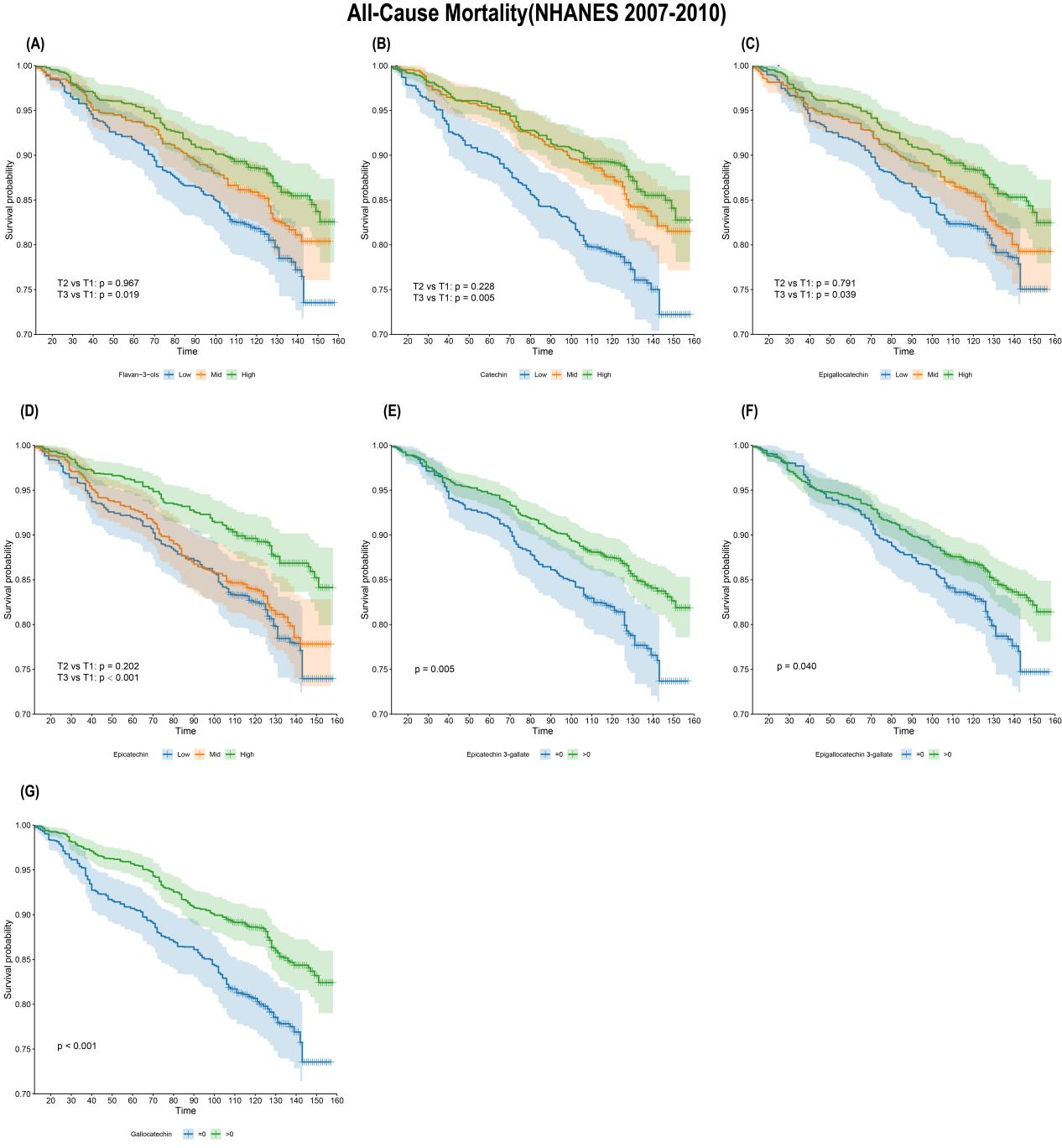


**Figure S3.** Kaplan-Meier Survival Analysis of Flavan-3-ols Intake and All-Cause Mortality(NHANES 2007-2010). (A)Flavan-3-ols and All-cause mortality; (B)C and All-cause mortality; (C)EGC and All-cause mortality; (D) EC and All-cause mortality; (E)ECG and All-cause mortality; (F)EGCG and All-cause mortality; (G)GC and All-cause mortality.

**References**

1. Pan J, Zhou Y, Pang N, Yang L. Dietary Niacin Intake and Mortality Among Individuals With Nonalcoholic Fatty Liver Disease. *JAMA Netw Open* (2024) 7:e2354277. doi: 10.1001/jamanetworkopen.2023.54277

2. Inker LA, Eneanya ND, Coresh J, Tighiouart H, Wang D, Sang Y, Crews DC, Doria A, Estrella MM, Froissart M, et al. New Creatinine- and Cystatin C–Based Equations to Estimate GFR without Race. *N Engl J Med* (2021) 385:1737–1749. doi: 10.1056/NEJMoa2102953
